# Artificial Dentures

**Published:** 1860-09

**Authors:** J. Allen


### ARTIFICIAL DENTURES.

BY DR. J. ALLEN.

Read before the American Dental Convention at Saratoga, August 7th, 1860.

In the construction of artificial dentures there are many important considerations that might be dwelt upon with interest would time permit, but on this occasion we will dwell mainly upon two leading points which lie at the threshold of this branch of our profession, for, to be successful in this department, we must begin right, and keep right, in order to come out right.

We will first consider the attainments necessary for him who constructs artificial dentures, for here is the starting point. And secondly the modes employed in their formation.

It is said there are five hundred causes for the stopping of a watch. If this be true, (and the books affirm it,) how many causes are there that would defeat a good practical result in forming a set of artificial teeth. From the taking of the impression through all the different stages of the work, to the final completion of a denture, various causes may occur, either of which might prove fatal to perfect success. In order to prevent a defeat from any of these causes, let us look for a moment at the acquirements necessary for him to possess, who is to reproduce those organs which nature with all her perfections had previously formed; for, whatever be the mode employed by man, he will have to learn that it is the hight of art to conceal art. This, together with practical utility, should be the great point aimed at in the construction of artificial dentures, and he who attains this end must be an artist in the true sense of the term. This requires thorough mental training and careful scientific research.

According to our best lexicographers, an artist is one who is skilled in the practice of some art, in which science and taste preside over the manual execution. It is thus that the artist is distinguished from the artizan; one copies from the works of nature in executing his designs, the other from the devices of man.

The artist finds resources in his own mind. There they are concealed from the world, and no human power but his own can bring them out of the storehouse in which they were coined, while the artizan works by fixed rules and principles which are transmitted from one generation to another, and serve as unerring guides for the mechanic, by means of which he is enabled to reproduce his fabrics ad infinitum, and all precisely alike, and each serve the purpose intended. A dentist who constructs artificial dentures as they should be, must possess both of the above acquirements in a high degree, for the great variety of cases that may occur in his practice will require the skill of the artist to conceive, and that of the artizan to execute his designs, in order to meet the various exigencies involved in his operations. He has no fixed forms or rules by which to be governed, for he encounters some new phase in each succeeding case. In one instance he has to insert teeth not more than the fourth of an inch in length, which even then appear too long; in another, those of an inch in length appear too short. Mrs. A. requires very large attachments to restore the sunken portions of her face, while Mrs. B. can scarcely bear the thinnest plate in her mouth without producing distortion.

The light and darker shades, the peculiar tint and tone of the teeth and gums, the size, form, position and expression of these organs, together with their perfect adaptation and harmony with the other features of the person for whom they are intended, require the finest powers of discrimination and manly execution, in order to conceal the art employed in forming them, which can now be done more perfectly than at any former period in the history of man. But to do all this the operator must possess a knowledge of several other branches of art and science, that he may bring to his aid such auxiliaries as are necessary to accomplish his purpose.

Metalurgy will claim his attention, that he may know the nature and properties of the various metals used, and the means of preparing them for dental purposes.

He must also understand something of mineralogy that he may be able to characterize, distinguish, classify and apportion the various minerals used in his profession, together with the art of converting them into artificial dentures, especially if he does continuous gum or block work. Our dental colleges and the community require of him a knowledge of anatomy, physiology, chemistry, and therapeutics, together with the principles and practice of dental surgery.

He must also possess mechanical skill which involves an insight into several of the trades, such as working the precious metals, modeling, moulding, etc., etc.; in short, he must be a sort of compound man, embodying several other branches of business in order to perfect his own.

All these form only the foundation of his professional career. The superstructure has yet to be built, which requires energy of character, in order to grapple successfully with the difficulties he will have to encounter; and perseverance equal to the task of overcoming them.

He also requires the mental capacity of a skillful tactician who can hold a sway over his patrons, that he may win and retain their confidence and esteem, however various their temperaments, views, or caprice. He must possess sufficient flexibility of mind to keep them all adroitly poised, so that each may add strength to his professional merit, and to crown all, he should be a perfect gentleman, with all the accomplishments that adorn his title.

With these acquirements we claim for him a position in the front rank of his profession. But a respondent says, that very many of those who construct artificial dentures do not possess all these qualifications; that they are not artists; that in the execution of their work their proximity to nature is not so near as to conceal the human agency employed; that their artificial work has not that graceful, natural, and life-like appearance which characterize the natural organs; that there is a want of proper tone, expression, adaptation, and practical utility, in part, if not all, of these essentials, and that their dentures exhibit only a stiff, mechanical appearance, that enables every beholder to spot them at once as artificial teeth, and hence the subordinate position of the mere mechanical dentist, whose claims to supremacy are but small, and whose merits are still less.

Alas! alas! this is all too true,—for such we do not claim an elevated position. But it does not follow that the well qualified should be placed upon the same level with those who form the lower ranks in this department. No, let those who occupy a higher ground stand firmly where they are, and not relinquish to their inferiors that branch which is of so much importance to the civilized world, as some of our best operators have done, especially in the larger cities.

Let these worthies stand at the helm, and take special cognizance of every new phase in their practice; let them examine with care all the points in each succeeding case, and devise such means as will best secure the ends sought to be attained; for with their experience and discrimination they can see the end in the beginning of their operations, and provide for all contingencies in due time, but the novice can not perceive them until too late for remedy. Let those who are below ascend to the higher ranks, bringing with them science, art and skill, gathering all along their pathway here a little and there a little, in order to fill up the great storehouse of knowledge from which success must flow. In reference to the various modes now in vogue, they all seem to have their place, and may be adopted under the different circumstances attending each particular case. When consulted, the operator should examine carefully and then decide as to what method will secure to the patient the greatest degree of practical utility, and personal gratification, and then advise accordingly, bearing in mind that whatever be the mode, skill, taste and judgement must preside over the operation, for there is no system so perfect as to render these unnecessary, and the farther the method is from yielding results true to nature, the harder it will be for the dentist to construct his work so as to elude detection in the mouth. In this respect we have been able to approach much nearer the acme of our ambition, by means of the continuous gum system, than any other with which we are familiar.

Objections are urged against every mode in use, some of them real, others speculative, but as time will not permit detail, I deem it proper to dwell more particularly upon what is called continuous gum work, although this term does not convey an adequate idea of the full embodiment of this system. As this style of work speaks for itself, nothing that we could say by way of eulogium would add to its merits, we will therefore notice the objections urged, by some to this method. It is said that dentures made in this way are too heavy. This objection is merely speculative, and has *no weight practically*, for with a good fitting plate, a patient cannot perceive the difference between a denture weighing five or fifteen pennyweights when in the mouth, for it will require several pounds, besides the weight of the teeth, to dislodge them. Another objector says they are too solid, they will not yield sufficiently when biting upon them, that they ought to have joints and crevices so as to spring in the mouth. This is an erroneous theory, for the natural gum under the base of the teeth will yield sufficiently for all practical purposes.

Mrs. W. says they are liable to break in a wash bowl or upon a marble slab or floor. The best remedy for this is to not let them fall. Do people continne to use pewter plates because china are liable to break if they fall? Shall we discard the use of watches because they are liable to similar accidents? But Dr. B. says if a tooth gets broken it can not be repaired. This is also an error; a single tooth or any number required can be re-inserted with comparatively little trouble to one who is accustomed to this style of work.

Dr. S. objects because the teeth clatter and make a noise when used. True, they have made considerable noise in the world, and will continue to make more. But the clattering should be stopped. This is owing to the lower plate being too wide and riding upon the muscles, ducts and glands which buoy up the lower denture and keep it afloat in the mouth; or, the teeth may be too long. To remedy this the plate should be made narrower or the teeth shorter.

Dr. D. asserts they are liable to break by use in the mouth. Does any other method fail? From long practical experience we *do know* that when continuous gum work is properly constructed the per cent. of breakage is far less than by the old methods.

We will now look at some of the reasons why this style of work fails. In most instances where this occurs they can be traced to some defect in the manipulations, or want of judgment in constructing the denture.

One of the common defects occurs in the soldering of the teeth to the plate. Platinum, unlike silver or gold, absorbs the solder commonly used, so that if the joints are not perfectly fitted the gold (used for solder) will become absorbed by the subsequent heatings into the platinum, and leaves no metallic union between the teeth and plate. To remedy this the inside rim or stays should be in positive contact with the base plate and then firmly soldered.

Another defect occurs from a want of experience with the furnace; a piece may be overbaked, or not baked hard enough, or not properly annealed, either of which would cause the body and gum to be brittle. To avoid this, the first bake should be scarcely glossed, the second a little harder, (but not perfectly smooth as though it were glazed,) and the last, or enamel heat should be a little higher than the preceding. This will give an even temper to the piece, and prevent crazening or cracking, if it be then properly annealed, which consists in leaving it in a heated muffle for several hours (say over night,) after which it is withdrawn from the muffle in which it was fused.

Another cause of failure arises from wrong articulations, the teeth being placed outside the alveolar ridge which throws an undue amount of leverage or strain upon the denture. To remedy this the upper teeth should be so arranged upon the plate as to stand perpendicularly upon the natural gum, and the pressure when the teeth are closed should rest upon the inner rather than the outer cusps of the molar and bicuspid teeth. In lower sets the back and side teeth should incline inward sufficiently to antagonize with the upper one, taking care not to infringe too closely upon the tongue. The above remarks pertain more particularly to full sets of teeth. There is also an apparent want of judgment on the part of some who construct artificial dentures, for in point of strength they make them as a mechanic would wheel-barrows, all alike, instead of which some cases require to be four times as strong as others. For example, if an upper artificial denture antagonizes with good natural back teeth below, the force of the jaws, in masticating will be far greater than if there are artificial teeth below. There are many modifying circumstances which may vary the necessary degrees of strength for a denture, which the operator should take into consideration, and then construct his work accordingly. In one instance a thin plate (say thirty-four) with light inside rim and but little body and gum would be amply sufficient; while another case would require a twenty-eight plate with a double or tripple inside rim with a corresponding portion of body and gum. *Let it be put down as a fixed fact, that this system is a correct one and that continuous gum work can be made strong enough to meet all the requirements of any artificial denture*, provided, good judgment and skill direct the manipulations.

But still there are those who have come to the sage conclusion that it will never do. So said those who opposed the theory of Gallileo, when he proclaimed to the world that the earth turned upon its axis, and although compelled to sign his recantation, he could not suppress the conviction which gave utterance to the ever memorable expression, "*but it does go.*"

So with continuous gum work, *it does go*, and ever will while artificial dentures continue to be made

---

*Coloring the Bones of a Fœtus by Mixing Madder with the Food of the Mother.*—At a recent meeting of the Academie des Sciences in Paris, M. Flourens, according to the *Gaz. Hebdom.*, presented a fœtus whose bones and teeth were all of a most beautiful red. The mother had been subjected to a diet mixed with madder during the last twenty-five days of gestation. The same phenomenon has frequently been observed in lower animals.

